# A recurrent homozygous *ACTN2* variant associated with core myopathy

**DOI:** 10.1007/s00401-021-02363-7

**Published:** 2021-09-01

**Authors:** Michio Inoue, Satoru Noguchi, Kyuto Sonehara, Keiko Nakamura-Shindo, Akira Taniguchi, Hiroyuki Kajikawa, Hisayoshi Nakamura, Keiko Ishikawa, Megumu Ogawa, Shinichiro Hayashi, Yukinori Okada, Satoshi Kuru, Aritoshi Iida, Ichizo Nishino

**Affiliations:** 1 Department of Neuromuscular Research, National Institute of Neuroscience, National Center of Neurology and Psychiatry, Tokyo, 187–8551 Japan; 2 Medical Genome Center, National Center of Neurology and Psychiatry, Tokyo, 187–8551 Japan; 3 Department of Statistical Genetics, Osaka University Graduate School of Medicine, Osaka, 565–0871 Japan; 4 Integrated Frontier Research for Medical Science Division, Institute for Open and Transdisciplinary Research Initiatives, Osaka University, Suita, 565–0871 Japan; 5 Department of Neurology, Toyama Prefectural Central Hospital, Toyama, 930–8550 Japan; 6 Department of Neurology, Mie University Graduate School of Medicine, Mie, 514–8507 Japan; 7 Department of Neurology, Suzuka Kaisei Hospital, Mie, 513–8505 Japan; 8 Laboratory of Statistical Immunology, Immunology Frontier Research Center (WPI-IFReC), Osaka University, Suita, 565–0871 Japan; 9 Department of Neurology, National Hospital Organization Suzuka Hospital, Mie, 513–9501 Japan; 10 Department of Neuromuscular Research, National Institute of Neuroscience, National Center of Neurology and Psychiatry, 4-1-1 Ogawahigashi, Kodaira, Tokyo, 187–8502 Japan

Recently, Lornage et al. reported a dominantly inherited myopathy associated with monoallelic variants in *ACTN2*, which is characterized clinically by weakness in distal and/or proximal muscles and pathologically by cores in myofibers [2]. Here, we report eight patients in three unrelated families with muscle weakness, core myopathy, and a biallelic variant in *ACTN2*.

Clinicopathological features of the patients are listed in Table 1. In Family 1, F1-II-6 (Fig. 1a) developed muscle weakness, predominantly in distal muscles from 32-years old. In Family 2, F2-IV-4 (Fig. 1b) had proximal muscle weakness in lower limbs and acquired left exotropia from 60-years old. The latter may be attributable to weakness of the extraocular muscles, as reported previously [2]. He had myocardial diastolic dysfunction and atrial fibrillation at 78-years-old. There was an apparent autosomal dominant family history. Other affected family members reported muscle weakness beginning in their 40 s to 60 s. In Family 3, F3-II-1 (Fig. 1c) developed limb muscle weakness from approximately 60-years-old. Muscle weakness was generalized, but there was asymmetric atrophy in the lower limbs (Fig. 2, online resource). Age at onset of these patients was similar to that of previously reported patients [3].

**Table 1:** Clinicopathological features of patients with homozygous c.1439A > G variants in *ACTN2* and in the previous reports

		Family 1	Family 2		Family 3	Lornage et al. [2]	Savarese et al. [3]
Individual		F1-II-6	F2-IV-4	F2-V-1	F3-II-1		
Variants in *ACTN2* (NM_001103)		c.1439A > G (p.Asn480Ser) homozygous				c.2180 T > G (p.Leu727Arg) heterozygous c.2194_2226del [p.(Ala732_Ile742del) heterozygous	c.1459 T > C (p.Cys487Arg) heterozygous c.392 T > C (p.Leu131Pro) heterozygous
Inheritance		Recessive				De novo	Dominant
Sex		Female	Male	Male	Female	Male and female	Female and male
Age at muscle biopsy		33	69	60	76	9 & 45, 19	
Onset		32	60	39	60 s	Early childhood	34–60
Muscle weakness		Distal to proximal lower limbs	Proximal lower limbs	Proximal > distal lower limbs	Proximal	Diffuse	Distal to proximal
Cardiac symptoms		No	Myocardial diastolic dysfunction (E/e' = 17.61), left atrial enlargement (left atrial dimension = 50.3 mm), atrial fibrillation	No	No	Cardiomegaly, heart failure	Ischemic heart disease and pacemaker, atrial flutter and left ventricular hypertrophy
Other features		Calf hypertrophy	External strabismus, left knee joint replacement, mild postural tremor	External strabismus, hammer toe, contracture of Achilles tendon, left knee joint replacement	Hypertension, diabetes mellitus	Facial muscle weakness, ophthalmoplegia, ptosis decreased respiratory insufficiency, contractures	Myalgia, asymmetric hypertrophy and atrophy of calf muscles and quadriceps, atrophy in both forearms
CK (U/L)		886	443	NA	181	Normal	Normal—5,000
Muscle pathology	Rimmed vacuoles	Yes	Yes	No	Yes	Yes	Yes
	Nemaline bodies	No	Yes	NA^a^	No	Yes	Yes
	Increased fibers with internal nuclei	Yes	Yes	Yes	Yes	Yes	Yes
	Multiple cores/lobulated fibers	Yes	Yes	NA ^a^	Yes	Yes	Yes
	Fiber type abnormality	Type 1 fiber predominance, type 2B fiber deficiency	Type 1 fiber predominance, type 1 fiber atrophy	NA ^a^	Type 1 fiber atrophy	Type 1 fiber predominance	Type 1 fiber predominance, myopathic-type grouping

*NA* not available

^**a**^Muscle pathology was not available except for hematoxylin and eosin staining

**Fig. 1:**
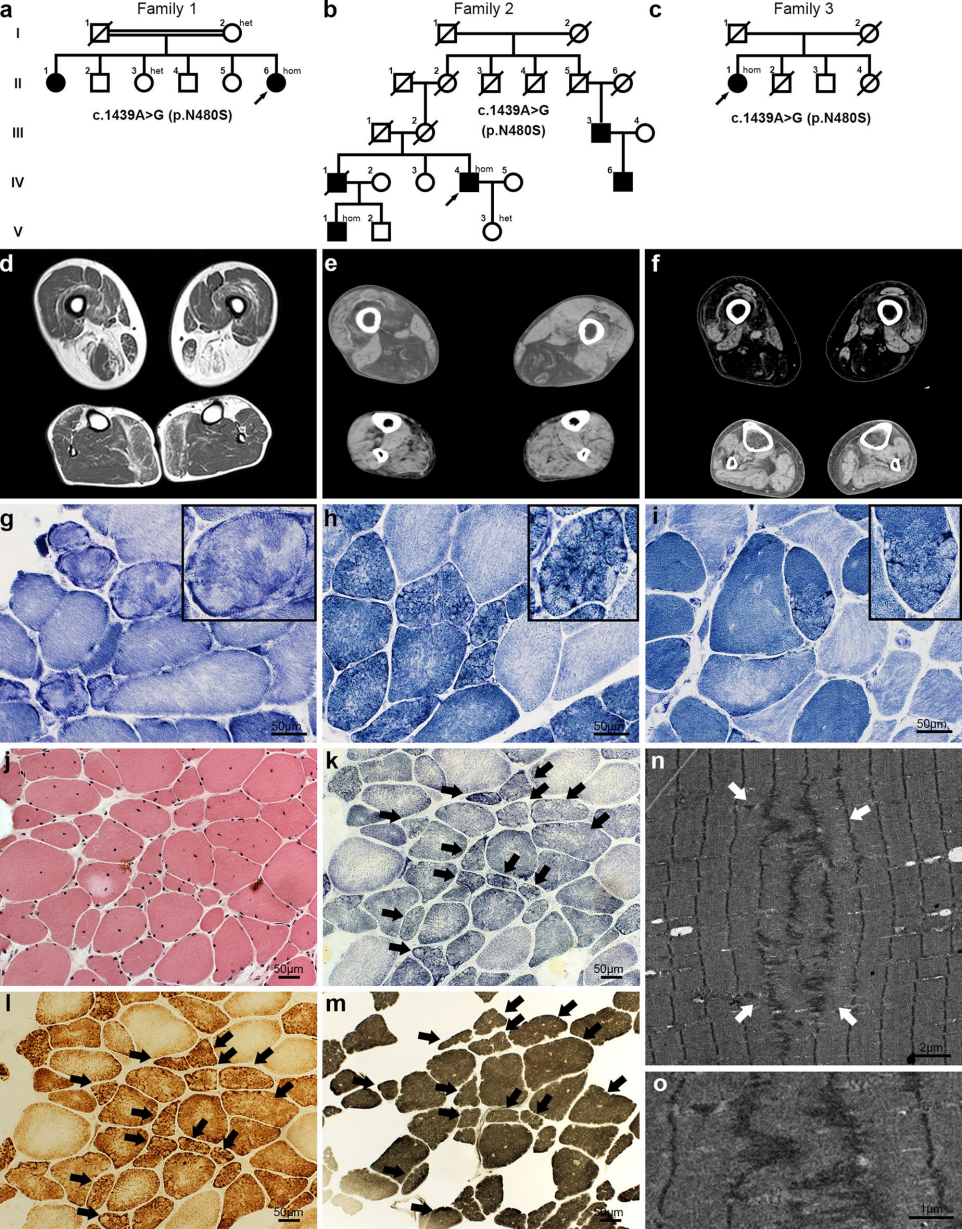
Patient muscle imaging and pathology. **a**–**c** Pedigrees in Family 1 (**a**), Family 2 (**b**), and Family 3 (**c**); individual genotypes are shown. **d**–**f** Thigh and calf muscle MRI (T1-weighted images) of F1-II-6 (**d**), CT of F2-IV-4 (**e**), and CT of F3-II-1 (**f**). **g–i** NADH- tetrazolium reductase staining of muscles from F1-II-6 (**g**), F2-IV-4 (**h**), and F3-II-1 (**i**); fibers with small cores (magnified images in the top right corners). **j**–**m** Histochemistry images of muscle serial sections from F2-IV-4. Minicores were detected on succinate dehydrogenase (**k**), cytochrome *c* oxidase (**l**), and myosin-ATPase at pH 4.0 (**m**) staining (arrows), but not hematoxylin & eosin (**j**). **n** Electron microscopic photograph of muscle from F2-V-1, showing Z-line with a zigzag appearance. **o** Magnified view of **n**

Muscle images showed fat replacement in the posterior compartment of the thigh, tibialis anterior, and medial head of the gastrocnemius (Fig. 1d–f). By contrast, the anterior compartment of the thigh, particularly the rectus femoris, sartorius, and gracilis, were spared. This pattern of muscle involvement is distinct from that previously described in patients with dominant variants in *ACTN2* [2, 3]. Muscle pathology in the three families was similar, showing moderate to marked variation in fiber size, scattered fibers with internal nuclei, and type 1 fiber predominance (Fig. 1g-j). Furthermore, many type 1 fibers had minicore-like structures and some of them looked like lobulated fibers as reported previously [2, 3]. These structures were visible not only by NADH-tetrazolium reductase staining but also by succinate dehydrogenase and cytochrome *c* oxidase staining, indicating an absence of mitochondria in the cores [4] (Fig. 1k–m). Rimmed vacuoles and nemaline bodies (Table 1 and Fig. 3, online resource) were also observed, similar to previous reports [2, 3].

Whole exome sequencing identified apparently homozygous *ACTN2* c.1439A > G (p.Asn480Ser) variants in affected individuals of all three families. This variant was present at extremely low frequency or not listed in public databases (Table 2, online resource). The highly conserved Asn480 residue is located in the second spectrin repeat and the pathogenicity of the p.Asn480Ser substitution was evaluated by in-silico prediction (Table 3, online resource). Although Family 2 appeared to exhibit dominant inheritance, analysis of whole exome sequencing data using XHMM demonstrated that the variant was homozygous in F2-IV-4 and F2-V-1 [1] (Fig. 4, online resource), suggesting possible pseudo-dominant inheritance pattern. Despite the complete co-segregation of the homozygosity of the variant with the disease (Fig. 1a-c), no stretches of identity-by-descent haplotypes were detected within the homozygous region, suggesting that the families do not share common ancestors (Fig. 4, online resource). Detailed experimental procedures are available online.

Cytoplasmic accumulation of TDP-43 and p62 was observed in patient muscles (F1-II-6 and F3-II-1) (Fig. 3, online resource). Actinin-2 accumulation was also observed in a similar pattern to nemaline bodies in a muscle serial section from individual F2-VI-4 (Fig. 3, online resource), suggesting that *ACTN2* p.Asn480Ser contributes to abnormality of Z-lines. Electron microscopy observation revealed Z-lines with a zigzag appearance becoming jagged Z-lines (Fig. 1n, o and Fig. 5, online resource), showing core structures, similar to the findings in dominant cases reported previously [2].

Alpha-actinin-2 protein was comparatively expressed in patient’s muscle to that in control (Fig. 6, online resource). In vitro behavioral analyses of alpha-actinin-2 with p.Asn480Ser indicated that this variant does not interfere homodimerization and intracellular localization of alpha-actinin-2 (Figs. 6–9, online resource), as previously shown for dominantly inherited mutants [2]. Moreover, in the human alpha-actinin-2 crystal structure, Asn480 contributes to serve hydrogen bonds with Asn469, building to the rod structure in the second spectrin repeat and Ser substitution may alter the interaction between two helices (Fig. 10, online resource), similar to Cys487, which is mutated in dominant myopathy [3]. This may explain the reason why the symptoms of the patients in this cohort were similar especially in the age of onset, asymmetric muscle atrophy, and cardiac symptoms to those of the patients with p.Cys487Arg (Table 1) [3]. Taken together, our data indicate that this variant may have a marginal effect on the function of alpha-actinin-2, which may explain its association with late-onset, relatively mild myopathy.

In addition to the patients with dominantly inherited *ACTN2* variants [2, 3], our data reveal that the Asn480Ser variant is hypomorphic and causes core myopathy in recessive mode. Further functional studies are required to elucidate the pathomechanism underlying this myopathy.

## Supplementary Information

Below is the link to the electronic supplementary material.

Supplementary file1 (DOCX 46958 KB)

## References

[1] Fromer M, Moran JL, Chambert K (2012). Discovery and statistical genotyping of copy-number variation from whole-exome sequencing depth. Am J Hum Genet.

[2] Lornage X, Romero NB, Grosgogeat CA (2019). ACTN2 mutations cause "multiple structured core disease" (MsCD). Acta Neuropathol.

[3] Savarese M, Palmio J, Poza JJ (2019). Actininopathy: a new muscular dystrophy caused by ACTN2 dominant mutations. Ann Neurol.

[4] Sewry CA, Wallgren-Pettersson C (2017). Myopathology in congenital myopathies. Neuropathol Appl Neurobiol.

