# Supplementary material for: A recurrent homozygous ACTN2 variant associated with core myopathy
Source: Acta Neuropathol. 2021 Sep 1;142(4):785–8. doi: 10.1007/s00401-021-02363-7 (PMC8423689; doi:10.1007/s00401-021-02363-7)
Supplement: Supplementary file 1 — Supplementary file1 (DOCX 46958 KB) [file 401_2021_2363_MOESM1_ESM.docx]

**Supplementary Information**

Article title: A recurrent homozygous *ACTN2* variant associated with core myopathy

Journal name: Acta Neuropathologica

Author names: Michio Inoue, Satoru Noguchi, Kyuto Sonehara, Keiko Nakamura-Shindo, Akira Taniguchi, Hiroyuki Kajikawa, Hisayoshi Nakamura, Keiko Ishikawa, Megumu Ogawa, Shinichiro Hayashi, Yukinori Okada, Satoshi Kuru, Aritoshi Iida, Ichizo Nishino

Affiliation and e-mail address of the corresponding author:

Satoru Noguchi

Department of Neuromuscular Research, National Institute of Neuroscience, National Center of Neurology and Psychiatry, 4-1-1 Ogawahigashi, Kodaira, Tokyo, 187–8502, Japan

E-mail address: noguchi@ncnp.go.jp

**Supplementary material**

**Figures and tables**


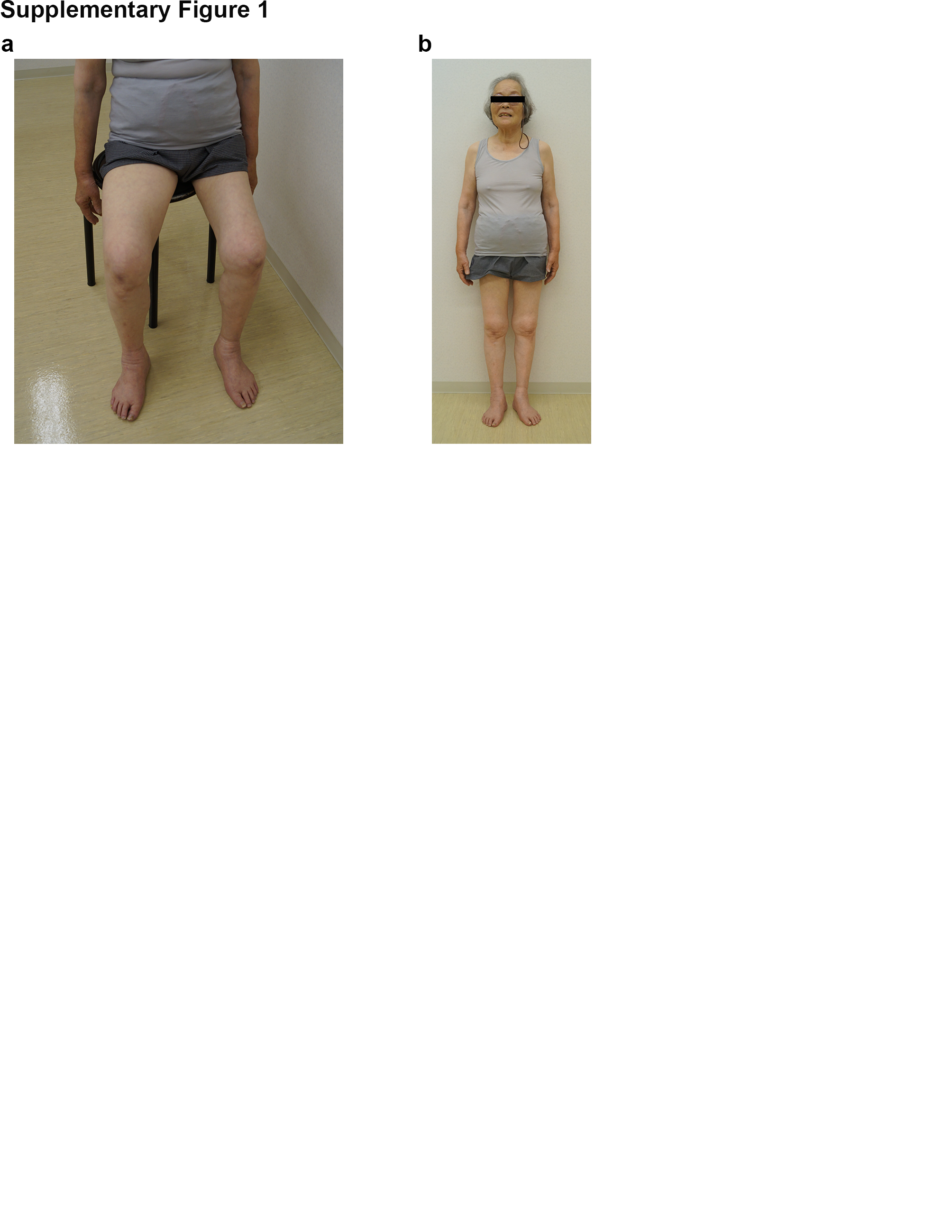


**Figure 2, online resource. Pictures of the patient (F3-II-1)**

**a** Asymmetric atrophy of the lower limbs. **b** Facial muscle involvement, atrophy of upper limbs or trunk muscles are not observed.


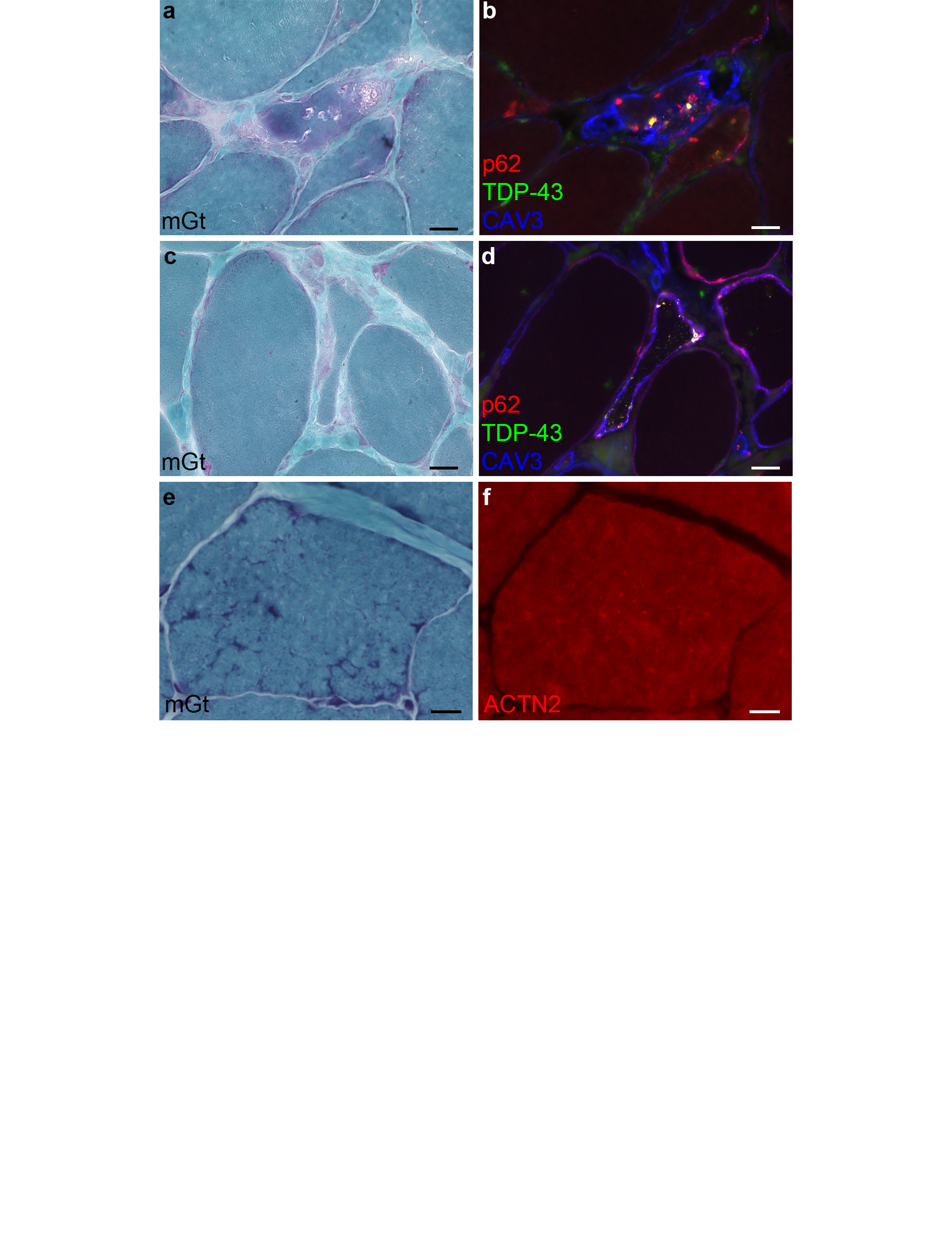


**Figure 3, online resource. Protein aggregates found in the patients’ muscles**

Muscle serial sections from F1-II-6 (**a, b**) and F3-II-1 (**c, d**). TDP-43 and p62 deposits are seen in atrophic fibers with rimmed vacuoles. Nemaline-like appearance on modified Gomori trichrome (**e**) and immunohistochemistry against alpha-actinin-2 (**f**) in the serial sections of the muscle from F2-IV-4. mGt, modified Gomori trichrome; CAV3, caveolin 3. Scale bars: a-d = 20 μm, and e,f = 10 μm.


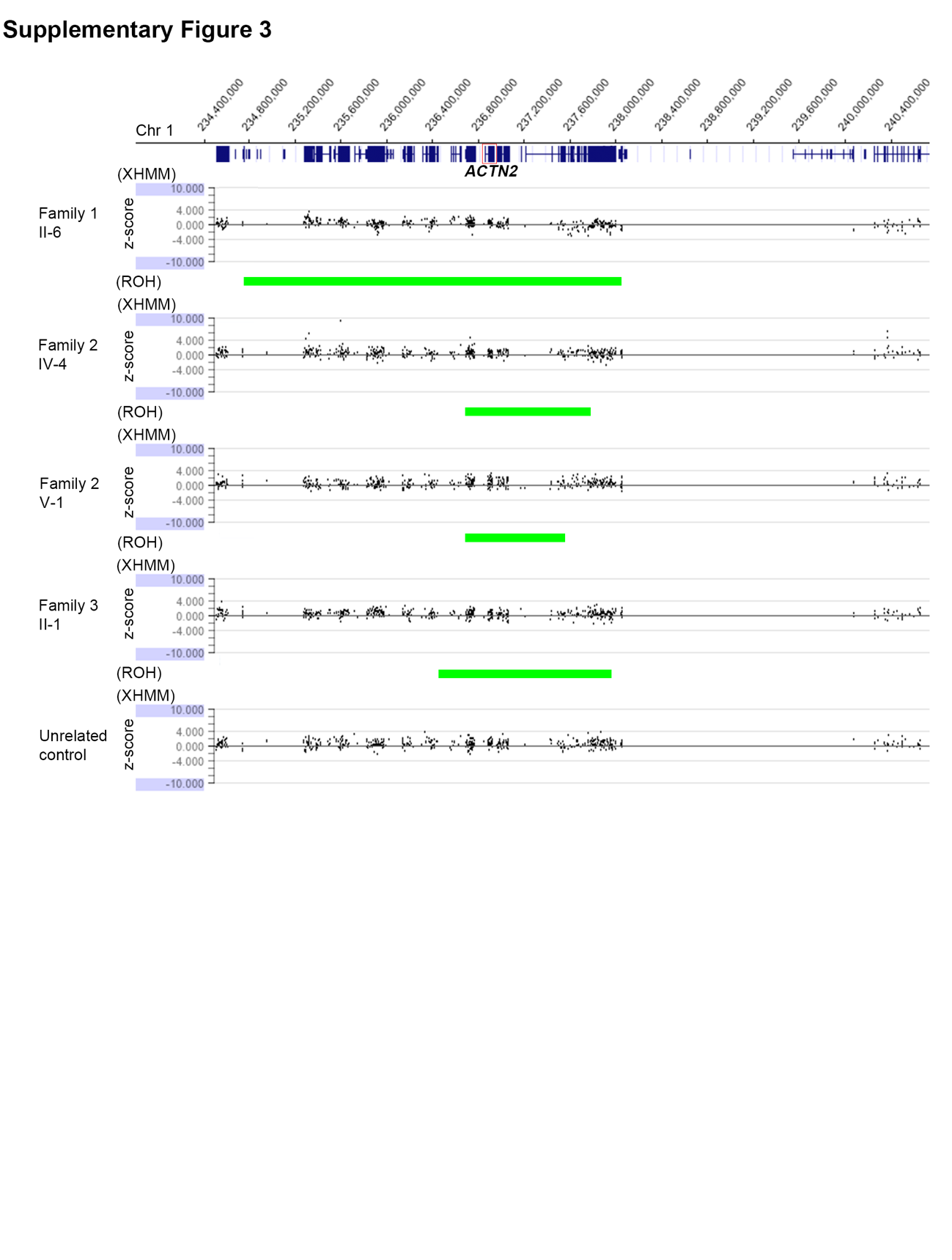


**Figure 4, online resource. No copy number variation by XHMM and runs of homozygosity of the patients**

In the results of copy number variation analyses by XHMM, the X-axis shows genomic position and the Y-axis indicates the z-score for each position. There are no apparent copy number losses involving *ACTN2.* Among all family members included in the genetic analysis, homozygosity for the variant completely co-segregated with disease. We detected runs of homozygosity including *ACTN2* in all affected, but not unaffected, family members ROH: Green bars, the regions with genetic homozygosity. ROHs covered whole *ACTN2* in all affected individuals. ROH, runs of homozygosity; XHMM, eXome Hidden Markov Model.


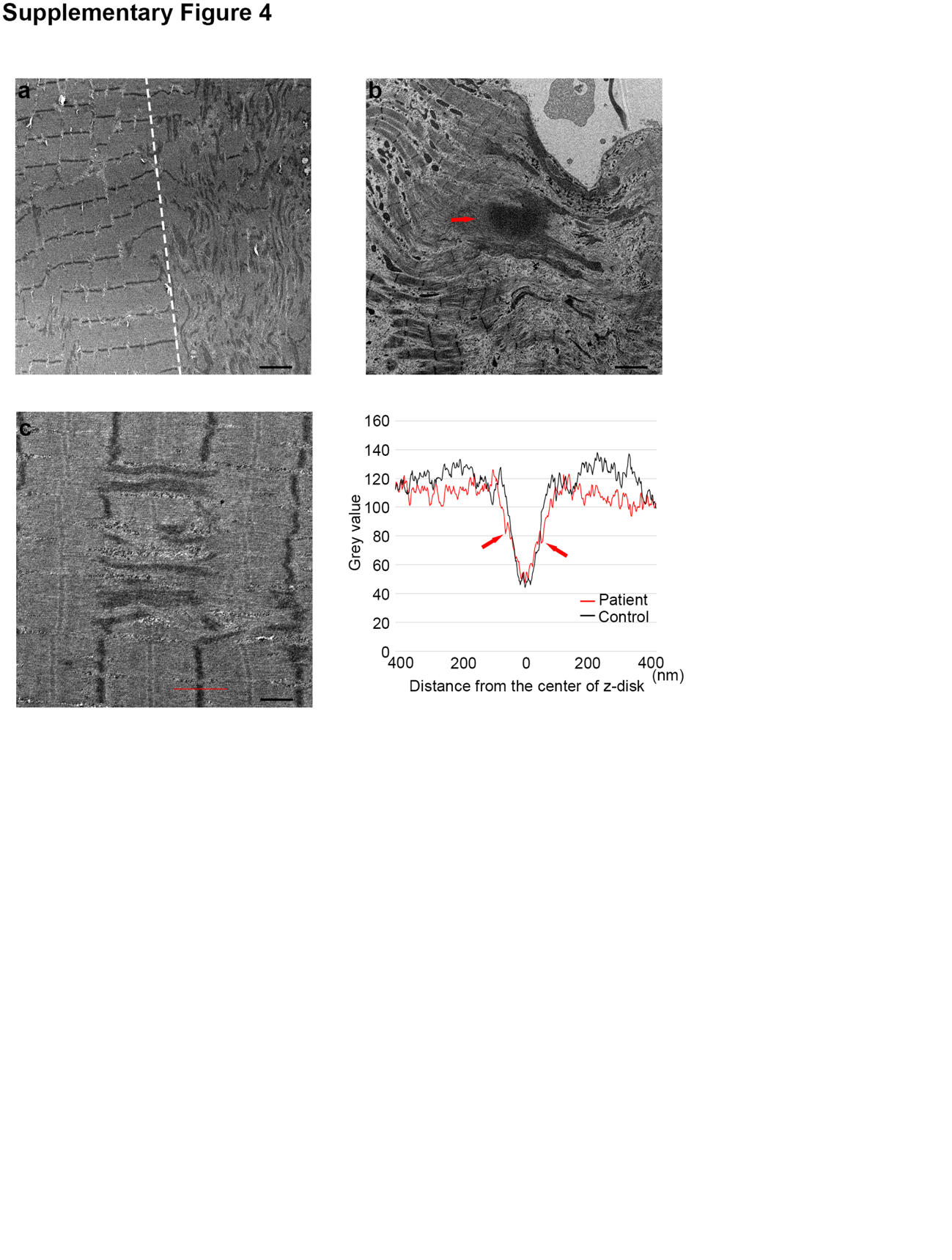
**Figure 5, online resource. Ultrastructural alterations in the patient’s muscle (F2-V-1)**

a Transition of zigzag Z-lines towards jagged Z-lines from left to right, showing core structures (right side of dash line). **b** Accumulation of dark material likely derived from Z-disk (arrow). **c** Disturbed sarcomeric structures. **d** line-plot profiles across Z-line in apparently non-disturbed sarcomeres as shown in red in c. The width of z-lines was enlarged with gradual drops in gray scale (arrows) to valley in patient’s muscle, suggesting blurred Z-disk even in non-disturbed sarcomeres. Scale bars = 2 μm.

**
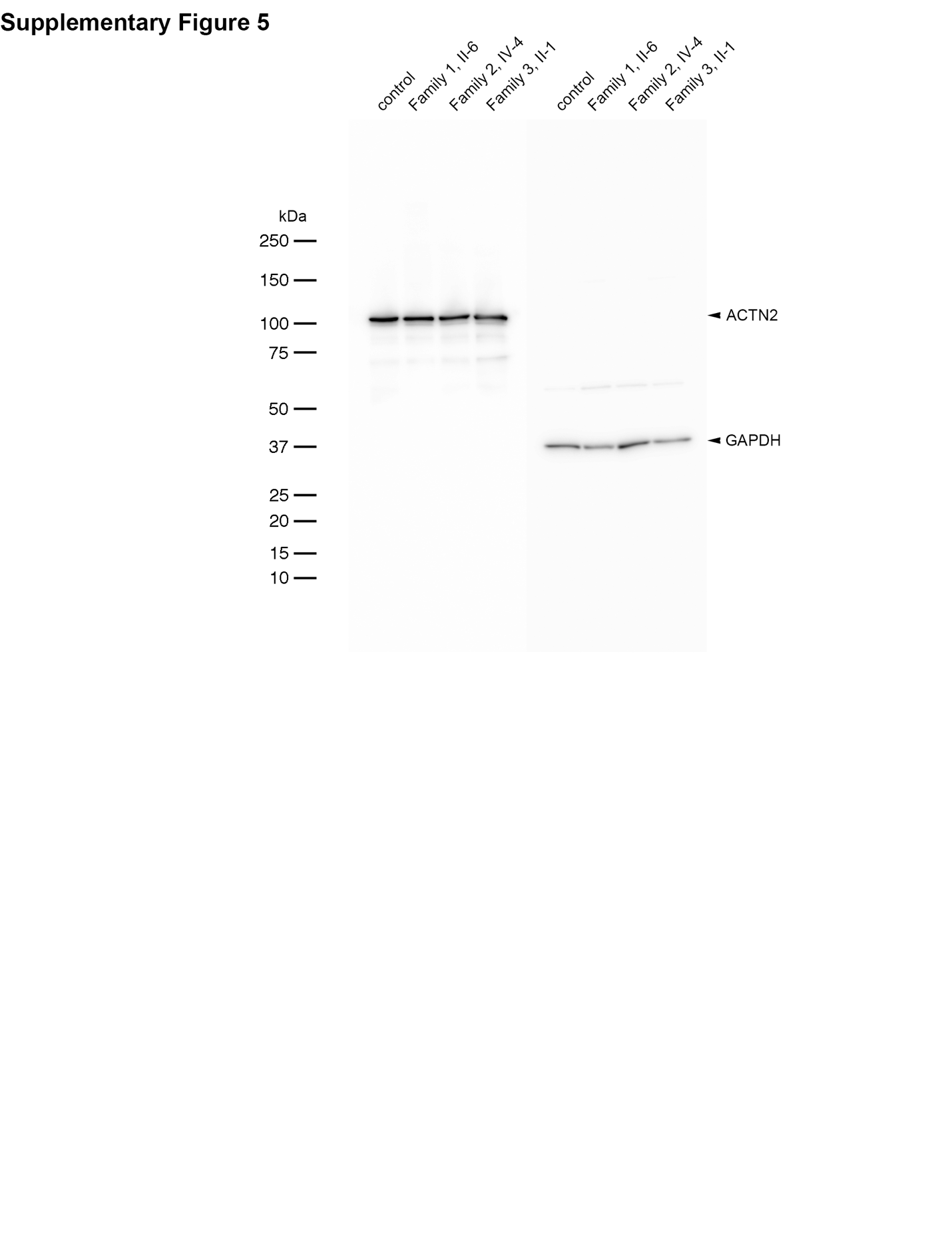
**

**Figure 6, online resource. Comparable expression of alpha-actinin-2 in Patients’ muscles**

Western blotting of muscle homogenates from F1-II-6, F2-IV-4, and F3-II-1 stained with anti-alpha-actinin-2 antibody and with GAPDH antibody as a loading control. A comparable band in both patient and age-matched control muscle samples were detected, demonstrating that the variant does not affect protein stability.


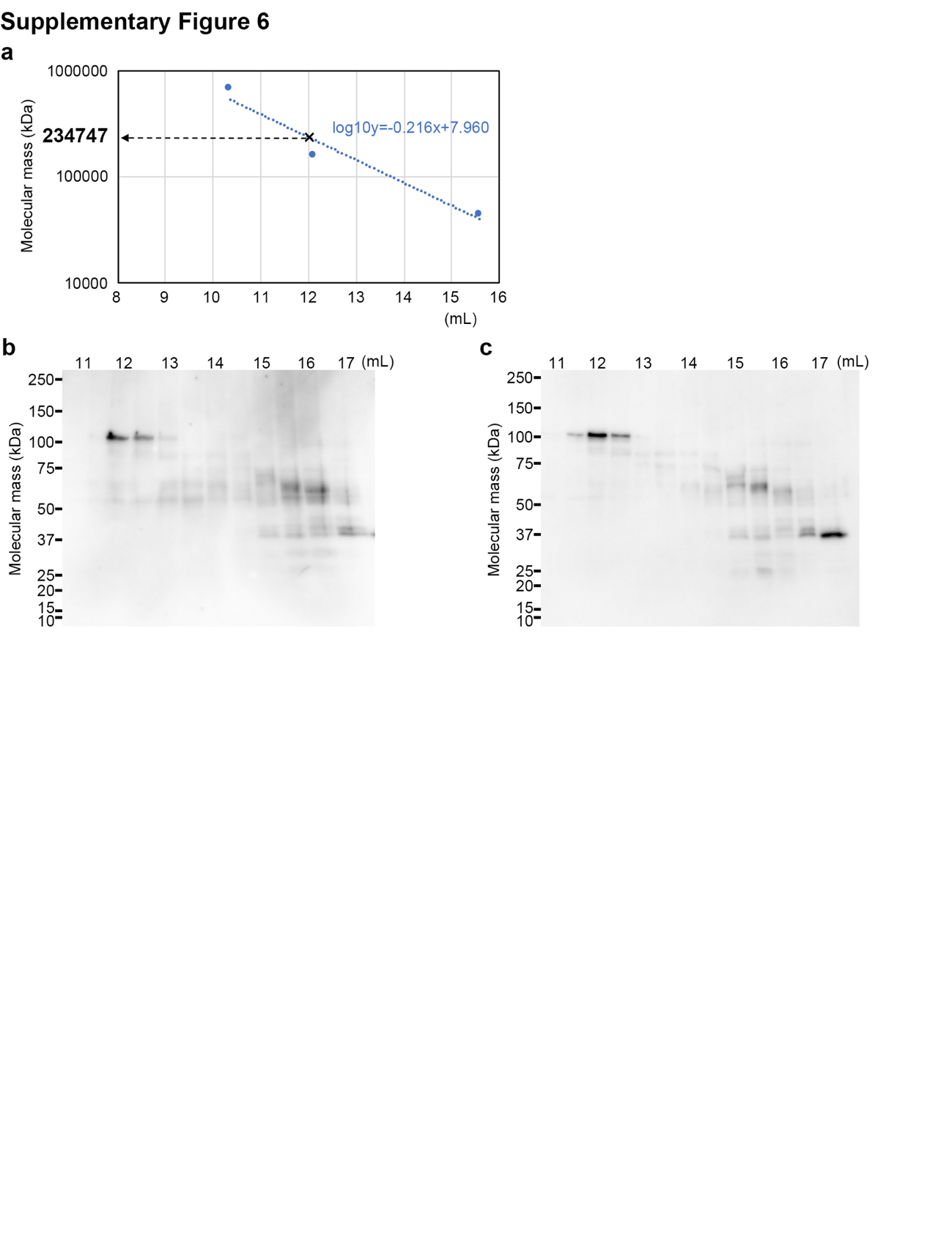


**Figure 7, online resource. Dimerization properties of wild-type and p.Asn480Ser alpha-actinin-2 on high-performance liquid chromatography**

**a.** Standard elution curve of marker proteins on gel filtration column with calculating formula. **b, c** Elution profiles of *in vitro* translated wild-type (**b**) and p.Asn480Ser (**c**) alpha-actinin-2 protein detected with PA-tag on SDS-PAGE. Both of wild-type and p.Asn480Ser alpha-actinin-2 at 120 kDa on SDS-PAGE were detected in fraction at 12 ml on HPLC and calculated as approximately 240 kDa (in bold in **a**) in native state showing both proteins to be dimers. This indicates that the variant does not interfere with its dimerization.

**
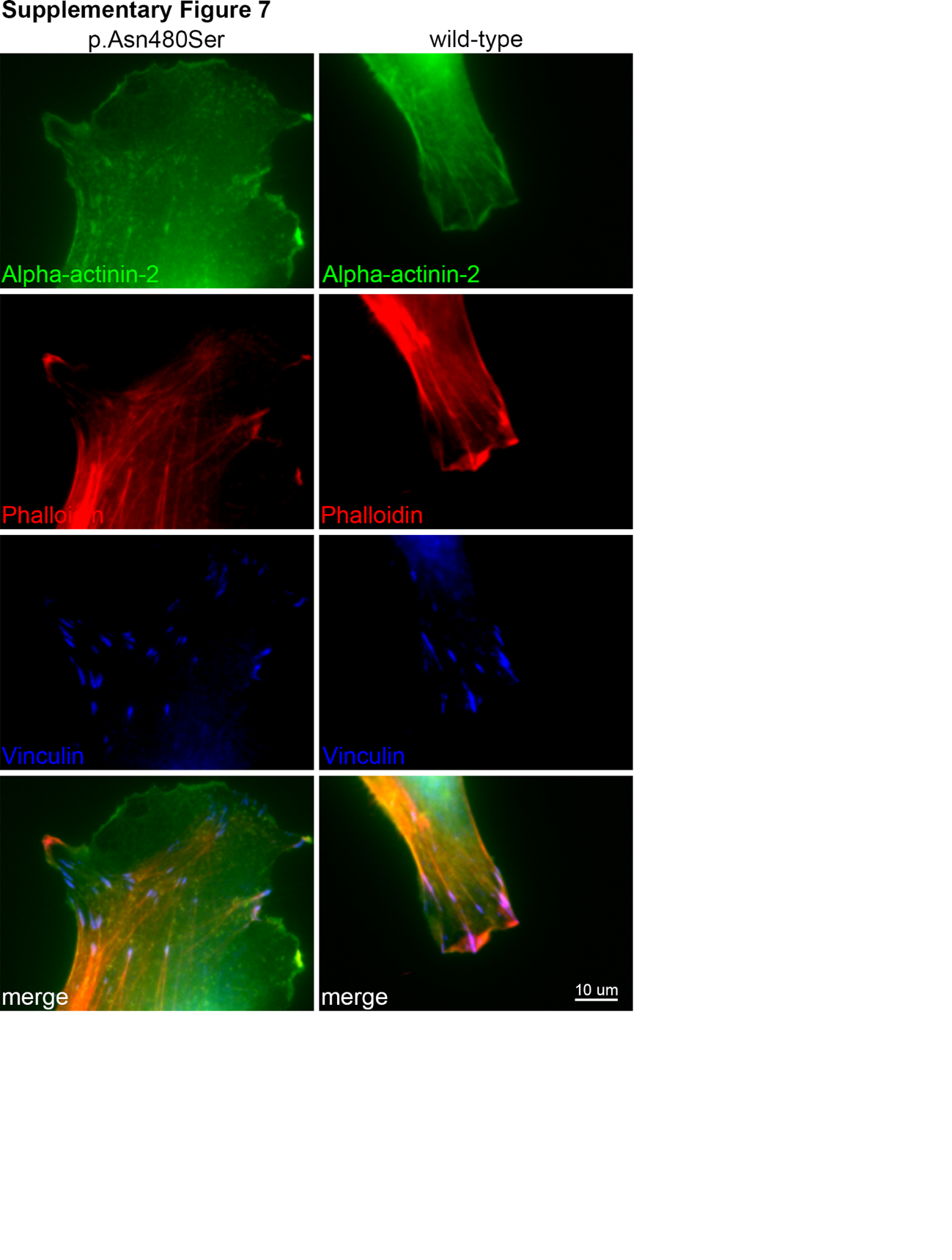
**

**Figure 8, online resource. Localization of mutant and wild-type alpha-actinin-2 on 10T1/2 fibroblasts.**

Both mutant (Asn480Ser) and wild-type EGFP- alpha-actinin-2 (Green) were recruited at focal adhesions. Vinculin and phalloidin are the markers for focal adhesions and stress fibers, respectively.

**
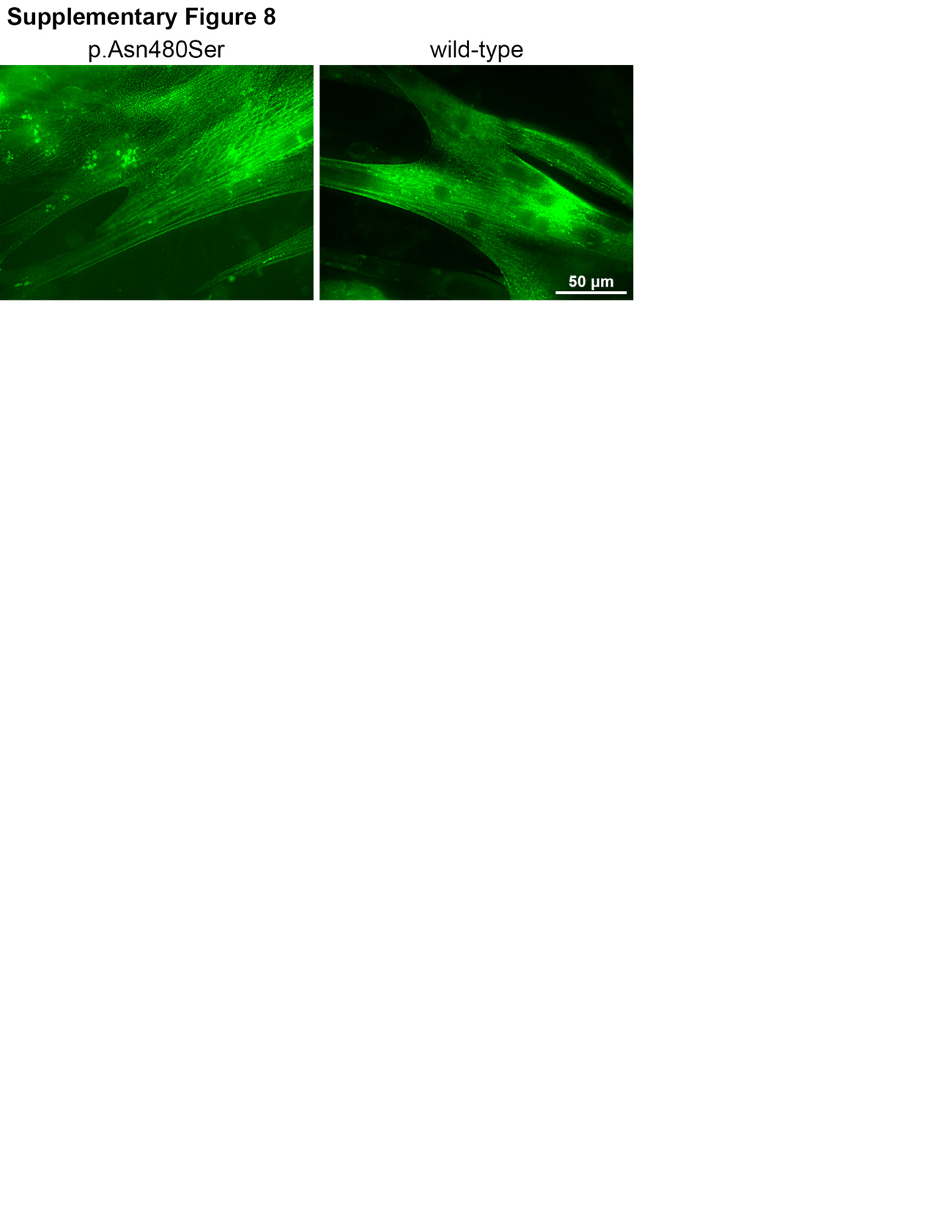
**

**Figure 9, online resource. Localization of wild and mutant** **alpha-actinin-2 in C2C12 myotubes**

Mutant (p.Asn480Ser) and wild-type EGFP-alpha-actinin-2 showed striation patterns in C2C12 myotubes on day 8 after induction of differentiation, suggesting that p.Asn480Ser does not influence the intracellular localization of alpha-actinin-2.

**
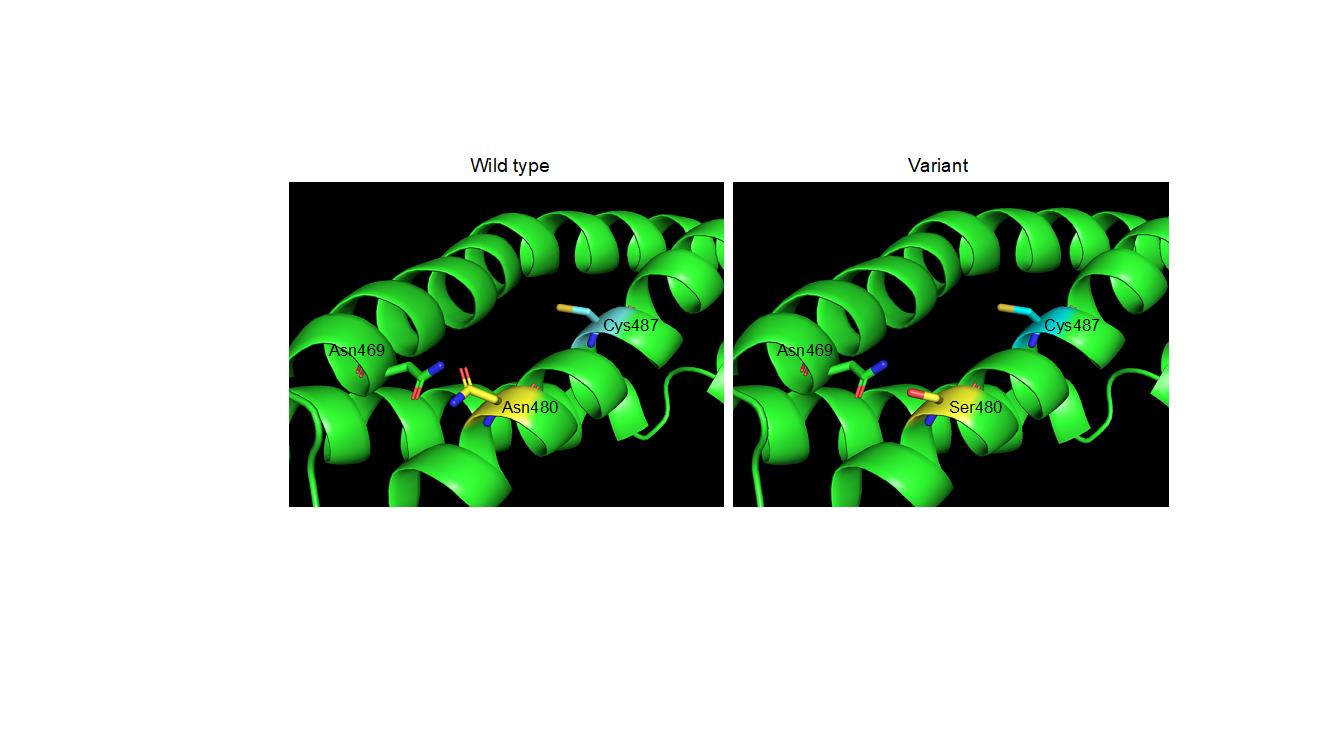
**

p.Asn480Ser

wild-type

**Figure 10, online resource. Structural models of SR2 region in wild type and mutated (p.Asn480Ser) alpha-actinin-2**

Three-dimensional model of SR2 based on the crystal structure in human alpha-actinin-2 (PDB ID: 4D1E). Asn480 (yellow) appears to closely interact with Asn469 in another alpha-helix via hydrogen bonds in left image (wild-type). In mutant alpha-actinin-2 (p.ASn480Ser), the replacement of Ser at 480 may reduce the interaction to Asn469 (right image). Cys487 is a residue mutated in previously reported dominantly-inherited patients [9], and the side chain of Cys487 is similarly directed toward the alpha-helix having Asn469.

**Table 2, online resource. Allele frequency of the c.1439A>G variant in *ACTN2* in the public databases**

|  | gnomAD^a^ | jMorp^b^ | HGVD^c^ | 1000G^d^ | ESP6500^e^ | HGMD^f^ |
| --- | --- | --- | --- | --- | --- | --- |
| c.1439A>G  (NM_001103.3) | .000003977 | 0.0001 | none | none | none | none |

^a^gnomAD, Genome Aggregation Database (<https://gnomad.broadinstitute.org/>)

^b^jMorp, Japanese Multi Omics Reference Panel (<https://jmorp.megabank.tohoku.ac.jp>)

^c^HGVD, Human Genetic Variation Database (<https://www.hgvd.genome.med.kyoto-u.ac.jp/>)

^d^1000G, 1000 Genomes (<http://www.internationalgenome.org/>)

^e^ESP6500, Exome Variant Server (<https://evs.gs.washington.edu>)

^f^HGMD, Human Gene Mutation Database (<http://www.hgmd.cf.ac.uk/>)

**Table 3, online resource. The pathogenicity of the p.Asn480Ser variant by *in silico* prediction**

|  | Mutation taster* | CADD** | LRT*** | Polyphen2**** |
| --- | --- | --- | --- | --- |
| c.1439A>G (p.Asn480Ser)  (NM_001103.3) | 0.81  Disease causing | 23.9 | 0.843  Deleterious | 0.693  Possibly damaging |

*Mutation Taster (<http://www.mutationtaster.org/>)

**CADD (<http://cadd.gs.washington.edu/>)

***LRT (<http://www.genetics.wustl.edu/jflab/lrt_query.html>)

****PolyPhen-2 (<http://genetics.bwh.harvard.edu/pph2/> )

**Patients and methods**

**Patients**

This study was approved by the institutional review board of the National Center of Neurology and Psychiatry and written informed consent were taken from the participants.

**Histological analyses**

We performed histological analyses as previously described [5]. Muscle samples were taken from the tibialis anterior of F1-II-6, rectus femoris of F2-IV-4, gastrocnemius of F2-V-1, and biceps brachii of F3-II-1, and then frozen in isopentane cooled in liquid nitrogen. A battery of histochemical analysis of serial frozen sections (10 μm thick), including hematoxylin & eosin, modified Gomori trichrome, nicotinamide adenine dinucleotide dehydrogenase-tetrazolium reductase (NADH-TR) staining, were prepared at the time of diagnosis. Immunohistochemical analysis was performed using the following primary antibodies: mouse anti-sarcomeric alpha actinin (Clone EA-53, Sigma-Aldrich, St.Louis, USA), rabbit anti-TDP-43 (10782-2-AP; Proteintech Group, Rosemont, USA), guinea pig anti-p62 (GP62-C-WBC; PROGEN, Heidelberg, Germany).

**Electron microscopy**

The gastrocnemius muscle of F2-V-1 was fixed in 2.5% glutaraldehyde and post- fixed with 2% osmium tetroxide. Ultrathin sections stained with uranyl acetate and lead citrate were observed under a transmission electron microscope (FEI, Hillsboro, USA). For analyzing the gray value around z-disk, we used sets of ultrastructural images which were acquired at 4800× magnification, and randomly selected 30 z-disks of the patient or the control samples. Using the software ImageJ [10], we evaluated the grey value of 400 pixels on each line segment, which crossed at right angles to a z-disk, and the midpoints of the line segments were located in the center line of z-disks.

**Whole exome sequencing**

We performed whole exome sequencing for affected individuals F1-II-6, F2-IV-4, F2-V-1, and F3-II-1 as well as for the unaffected 9 individuals F1-I-2, F1-II-3, F2-V-3 as previously described [1, 3, 4]. Genomic DNA was isolated from peripheral blood lymphocytes except for F1-II-6 (from a muscle specimen) using standard techniques. We confirmed the identified variants by Sanger sequencing.

**Nonparametric Identity-by-Descent Mapping**

We pruned the surrounding genotype data of the common variants (with minor allele frequency ≥1% in the general Japanese population) [7] using NBDC Human Database (https://humandbs.biosciencedbc.jp/, JGAD00000000220) with PLINK software (http://pngu.mgh.harvard.edu/,purcell/plink/), and assessed the runs of homozygosity (ROH) and identity-by-descent (IBD) status shared among the affected and unaffected individuals, based on the nonparametric IBD mapping method of single nucleotide polymorphism streak [6].

**Copy number variation analysis**

Copy number variations were evaluated using whole exome sequencing data with the eXome Hidden Mrkov Model (XHMM) algorithm as previously described [2]. The principal-component analysis-normalized and -filtered z-scores for the exome read depth were obtained. SignalMap Software v2.0 (Roche Diagnostics Corporation, Indianapolis, USA) was used for visualization.

**In vitro translation, and high-performance liquid chromatography (HPLC)**

PA-tagged alpha-actinin-2 proteins were synthesized using an in vitro transcription and translation system as described by the manufacturer (NUProtein, Kobe, Japan). Dimerization was determined by HPLC gel filtration. HPLC was carried out on a Superose 6 column (GE Healthcare, Amersham, UK), eluted with phosphate buffer saline at 1 ml/min. Equal amounts of each fraction were eluted and subjected to SDS-PAGE and Western blotting using rat anti-PA tag (NZ­1, FUJIFILM Wako Pure Chemical Corporation, Osaka, Japan) .

**Localization of mutated alpha-actinin-2 protein in10T1/2 fibroblasts and C2C12 myotubes**

The construction of wild-type and mutant human alpha-actinin-2 ORF into the expression vector, pEGFP-C1 (Clontech Laboratories, Mountain View, USA) was described previously [8]. The mutation was introduced by site-directed mutagenesis using the QuikChange II kit (Strategene, San Diego, USA). We transfected the plasmids into 10T1/2 cells and C2C12 cells with using Lipofectamine and PLUS reagent (Thermo Fisher Scientific, Waltham, USA), and fixed cells at 24 h after transfection for 10T1/2 cells and at 8 days after myogenic induction. Cells were fixed in 4% paraformaldehyde (PFA) for 15 minutes and permeabilized with 0.25%Triton X-100 in PBS. The antibody against mouse anti-vinculin (Clone VIN-11-5; Sigma-Aldrich) and phalloidin-Alexa 568 (Thermo Fisher Scientific) for identification of focal adhesion and stress fibers, respectively were used. Images were obtained on a Keyence CCD camera (Keyence, Osaka, Japan).

**Structure modeling**

Three-dimensional structural model were visualized in PyMOL based on the crystal structure of the human alpha-actinin-2 (Protein Data Bank ID: 4D1E).

**References**

1 Endo Y, Noguchi S, Hara Y et al (2015) Dominant mutations in ORAI1 cause tubular aggregate myopathy with hypocalcemia via constitutive activation of store-operated Ca(2)(+) channels. Hum Mol Genet 24:637-648

2 Fromer M, Jennifer, Chambert K et al (2012) Discovery and Statistical Genotyping of Copy-Number Variation from Whole-Exome Sequencing Depth. Am J Hum Genet 91:597-607

3 Iida A, Takano K, Takeshita E et al (2019) A novel PAK3 pathogenic variant identified in two siblings from a Japanese family with X-linked intellectual disability: case report and review of the literature. Molecular Case Studies 5:a003988

4 Inoue M, Uchino S, Iida A et al (2019) COX6A2 variants cause a muscle-specific cytochrome c oxidase deficiency. Ann Neurol 86:193-202

5 Malicdan MC, Noguchi S, Nishino I (2009) Monitoring autophagy in muscle diseases. Methods Enzymol 453:379-396

6 Okada Y, Diogo D, Greenberg JD et al (2014) Integration of sequence data from a Consanguineous family with genetic data from an outbred population identifies PLB1 as a candidate rheumatoid arthritis risk gene. PLoS One 9:e87645

7 Okada Y, Momozawa Y, Sakaue S et al (2018) Deep whole-genome sequencing reveals recent selection signatures linked to evolution and disease risk of Japanese. Nat Commun 9:1631

8 Sambrook J, Fritsch EF, Maniatis T (1989) Molecular cloning: a laboratory manual. Cold Spring Harbor Laboratory Press, City

9 Savarese M, Palmio J, Poza JJ et al (2019) Actininopathy: A new muscular dystrophy caused by ACTN2 dominant mutations. Ann Neurol 85:899-906

10 Schneider CA, Rasband WS, Eliceiri KW (2012) NIH Image to ImageJ: 25 years of image analysis. Nat Methods 9:671-675
